# Fast and slow thinking in distressing delusions: A review of the literature and implications for targeted therapy

**DOI:** 10.1016/j.schres.2017.08.045

**Published:** 2019-01

**Authors:** Thomas Ward, Philippa A. Garety

**Affiliations:** King's College London, Institute of Psychiatry, Psychology & Neuroscience, Department of Psychology, United Kingdom; South London and Maudsley NHS Foundation Trust, United Kingdom

**Keywords:** Paranoia, Persecutory, Dual process, Belief flexibility, Jumping to conclusions, Bias Against Disconfirmatory Evidence (BADE), mHealth, eHealth, Digital therapy

## Abstract

The recent literature on reasoning biases in psychosis and delusions is reviewed. The state-of-the-art knowledge from systematic reviews and meta-analyses on the evidence for jumping to conclusions is briefly summarised, before a fuller discussion of the more recent empirical literature on belief flexibility as applied to delusions. The methodology and evidence in relation to studies of belief flexibility and the Bias Against Disconfirmatory Evidence (BADE) across the delusional continuum will be critically appraised, and implications drawn for improving cognitive therapy. It will be proposed that dual process models of reasoning, which Kahneman (Kahneman, 2011) popularised as ‘fast and slow thinking’, provide a useful theoretical framework for integrating further research and informing clinical practice. The emergence of therapies which specifically target fast and slow thinking in people with distressing delusions will be described.

## 1 Introduction

Cognitive models of psychosis (Freeman et al., 2002, Garety et al., 2007, Garety et al., 2001, Morrison, 2001) propose that beliefs and appraisals play a central role in determining the clinical consequence of psychotic experiences. The way in which individuals make sense of, and respond to, anomalous experiences can determine whether they remain benign (even life-enhancing) or alternatively result in beliefs which are distressing and lead to impairment and a need for clinical care (Garety and Hardy, 2017, Peters et al., 2016). This paper presents key research findings pertaining to reasoning biases, delusional beliefs and psychosis. The state-of-the-art knowledge from systematic reviews and meta-analyses on the evidence for jumping to conclusions is briefly summarised, before a fuller discussion of the emergent empirical literature on belief flexibility as applied to delusions. Different ways in which the construct of belief flexibility has been studied are presented, highlighting a recent rapprochement with other well-established theoretical domains; specifically ‘dual-process’ models of reasoning which Daniel Kahneman popularised as ‘fast and slow thinking’ (Kahneman, 2011). We argue that a greater conceptual clarity in the cognitive operations underpinning important reasoning biases will facilitate theory refinement and the development of more effective targeted treatments for psychosis. SlowMo, a novel brief digital intervention for fears of harm from others is presented as a recent example of a treatment approach that targets reasoning biases as key maintenance factors in paranoia; an example of a wave of causal-interventionist approaches to the treatment of psychosis.

## 2 Jumping to conclusions

A tendency in people with psychosis to use fewer data to reach a decision is posited to contribute to delusion formation and persistence; thus, we have proposed that anomalous or ambiguous information is rapidly appraised and a delusional conclusion drawn on the basis of limited evidence, and without a thorough consideration of alternatives or a review of the evidence (Garety and Freeman, 2013). Systematic reviews and meta-analyses demonstrate a large and consistent evidence base in over 50 studies, in which the clear majority show that individuals with delusions and psychosis make decisions on the basis of limited evidence in probabilistic reasoning tasks; the so-called ‘jump-to-conclusions’ (JTC) data-gathering bias (Dudley et al., 2016, Garety and Freeman, 2013, So et al., 2016). Recently, researchers have also addressed the question as to whether this consistently replicated JTC association is specific to delusions or a feature of psychosis more generally. This is important both theoretically and clinically, since finding specificity to delusions supports the proposition that this reasoning bias contributes directly to the way psychotic experiences are appraised, increasing the likelihood of delusion occurrence. A narrative review of 61 studies of JTC by Garety and Freeman (2013) concluded that the JTC bias is likely to be specifically associated with delusions. However, given issues of study heterogeneity and sample selection, meta-analytic approaches may assist further in attempting to resolve this question. An early meta-analysis (Fine et al., 2007) reported a reliable association between JTC and delusions. In contrast, So et al. (2016) reported that due to lack of suitable, sufficiently powered group comparisons (i.e. comparison of JTC in groups with a diagnosis of schizophrenia with vs. without delusions) they could only conclude that “JTC bias is consistently evident in psychotic groups with varied symptom profiles” (p. 161). JTC was however not associated with other psychiatric diagnoses (notably depression) suggesting that it is not a transdiagnostic process. In another recent large-scale meta-analysis, Dudley et al. (2016) replicated the key conclusion of So et al. (2016), reporting clear evidence that people with psychosis make decisions on the basis of less information, with the odds of JTC in psychosis being between 4 and 6 times higher than in healthy participants and participants with nonpsychotic mental health problems, respectively. These authors also included an analysis of samples split into those with versus without delusions and concluded that JTC bias was linked to a greater probability of delusion occurrence in psychosis (k = 14, N = 770, OR 1.52, 95% CI 1.12, 2.05). Another recent meta-analysis (McLean et al., 2016) provided further evidence that groups with a diagnosis of schizophrenia with current delusions showed more JTC than those without (with a small effect size). In addition, it has been proposed that JTC is a trait representing vulnerability to delusions (Garety and Freeman, 2013, Dudley et al., 2016). The grounds for this are that the JTC bias, in attenuated form, has also been observed in those recovered from delusions (Peters et al., 2006), is associated with delusional thinking in the general population (Colbert and Peters, 2002, Freeman et al., 2008, Van Dael et al., 2006) and observed in those with an at high risk mental state and other at risk groups, e.g. relatives (Broome et al., 2007, Van Dael et al., 2006).

In summary, recent meta-analyses have now definitively established the association between JTC and psychosis. There is also evidence that this applies to risk of psychosis, predicts outcome in response to treatment and there is overall moderate-strong support for the specificity of JTC to delusions. Taken together these provide converging evidence indicating that JTC plays a causal role in delusion development and maintenance and therefore represents a target for both prevention and treatment strategies. However, it should be noted that while some longitudinal studies were included in the recent meta-analyses of Dudley et al. (2016) and McLean et al. (2016), both suggest that further work demonstrating that JTC co-varies with delusions over time in schizophrenia is required to establish a causal relationship conclusively.

## 3 Belief flexibility: 3 strands of investigation

Early work on the JTC bias has led to investigation into the construct of belief flexibility, a term referring to the degree to which a person demonstrates flexibility regarding a strongly held (delusional) belief. While JTC has been viewed as a data-gathering bias, the construct of belief flexibility can be viewed as a more complex meta-cognitive (higher order) reasoning construct. It involves an individual's ability to disengage from (‘decouple’) a strongly held (delusional) belief, once formed, in order to engage in further cognitive operations involved in making judgements under conditions of uncertainty: considering the possibility of being mistaken; reviewing the main belief in light of newer evidence/information (evidence integration); and generating and considering other alternatives (Fischhoff and Beythmarom, 1983, Hemsley and Garety, 1986). Belief flexibility in the context of psychosis has been examined in three main ways: the direct assessment of flexibility in reasoning about delusions; delusion-neutral tasks assessing a postulated Bias Against Disconfirmatory evidence; and dual process models of reasoning. We will consider each in turn.

1. Belief Flexibility as reasoning about delusions. Early work conducted by Garety and colleagues emerged from the development of a clinical research assessment of strongly held (delusional) beliefs; The Maudsley Assessment of Delusions Schedule (Wessely et al., 1993). While this work was influenced by earlier psychological theory in the area of decision making under uncertainty (Fischhoff and Beythmarom, 1983, Hemsley and Garety, 1986, Tversky and Kahneman, 1974), the MADS was designed to provide a comprehensive assessment of delusions and the ways in which individuals reasoned about their psychotic experiences and beliefs. From this early work, together with the development of the Explanations of Experiences (EoE) interview assessing Alternative Explanations (Freeman et al., 2004), we defined belief flexibility as the metacognitive capacity of reflecting on one's own beliefs, changing them in the light of reflection and evidence, and generating and considering alternatives (Garety et al., 2005). The assessment typically comprises 1) accepting the possibility of being mistaken (PM) 2) the ability to identify an alternative explanation (AE) to ones' own (delusional) belief, and 3) changing conviction in response to a hypothetical contradictory scenario (RTHC) (Garety et al., 2005, So et al., 2012).
  The literature on belief flexibility and delusions is more recent and less well developed than that for JTC, and accordingly a smaller number of empirical studies using this approach are reported. Belief flexibility (given its origins) has been discussed almost exclusively in the context of reasoning about delusional beliefs (although see Colbert et al., 2010 and Ward et al., 2017 for exceptions). Lack of belief flexibility is commonly reported in people with delusions, with rates of inflexibility on the commonly used PM item (MADS; Wessely et al., 1993) typically around 50% of people (Garety et al., 2005, So et al., 2012). Alternative explanations are found in around a quarter of people while absence of AE is associated with more anomalous experiences and higher JTC (Freeman et al., 2004), suggesting a route by which these processes interact in the development of a delusional belief. Exploratory factor analysis has confirmed that the three items yield a stable factor (So et al., 2012). However from a theoretical perspective, responses potentially involve different underlying cognitive operations (for example accepting the possibility of being mistaken requires ‘decoupling’ from the belief while generating an alternative explanation requires additional idea generation, involving ‘mental simulation’ and ‘holding in mind’ of a dual representation). One investigation which examined belief flexibility biases in people with persecutory and with grandiose delusions, found that while common in association with both delusion types, they are more strongly associated with grandiose delusions (Garety et al., 2013b). These findings suggest relationships between belief flexibility and emotional processes.
2. The Bias against Disconfirmatory Evidence (BADE; Moritz and Woodward, 2006, Woodward et al., 2006) represents a related but somewhat narrower construct than belief flexibility as defined above, in that it concerns a hypothesised bias in the evaluation of disconfirmatory evidence. The rationale for the development of an assessment of BADE (Woodward et al., 2006) was that the other key reasoning biases proposed for delusional beliefs (JTC, attributional and Theory of Mind biases) had been demonstrated with delusion-neutral material and therefore separated from the symptom itself, while belief flexibility (as assessed by MADS), by definition, involves reasoning about delusions. The BADE task was therefore developed to examine empirically whether people with delusions exhibit a cognitive bias in which they neglect disconfirmatory evidence (BADE) for their beliefs – and whether this occurs with non-delusion related content. The methodology of the BADE task has been subject to significant variability over time (both in terms of presentation of materials and calculation of key dependent variables). Most typically however, assessment of BADE involves an ambiguous delusion-neutral scenario (verbal or pictorial) which is then sequentially disambiguated. A number of interpretations are presented and rated for plausibility (typically separated into true, absurd and lure (emotional or neutral) interpretations). Evidence of BADE is usually defined as the lower reduction in plausibility of the lure items over time (as these initially plausible interpretations become disconfirmed in stages) in those with psychosis when compared to a comparison (healthy or other psychiatric control) group.
  An association between BADE and a diagnosis of schizophrenia has been consistently replicated, in empirical studies in comparison to both healthy controls and other psychiatric (mostly OCD) groups. In a further small group of studies in the general population, BADE has been significantly associated with subclinical delusional ideation (Menon et al., 2013, Zawadzki et al., 2012) using the PDI (Peters et al., 1999), and found in high vs. low schizoptypy student groups in some (Buchy et al., 2007) but not all (Orenes et al., 2012) studies. There have been recent calls to standardise the methodology and adopt dependent variables drawn from factor analysis of the entire set of plausibility ratings (Sanford et al., 2014, Speechley et al., 2012) rather than the a priori computation of BADE (which has varied considerably across studies). The most recent of these factor analytic studies (Sanford et al., 2014), including a sample of 43 patients with delusions proposed two components underpinning performance on the BADE task: ‘evidence integration’ (the degree to which disambiguating information has been used) and ‘conservatism’ (a reduced willingness to provide high plausibility ratings when justified). The study found that only evidence integration differed between severely delusional patients and the other groups. In contrast with a previous distinction between BADE (down-ratings of plausibility on lure items) and a Bias against Confirmatory Evidence (BACE; uprating of plausibility on true interpretation), response to both lure and true items loaded on to the ‘Evidence integration’ component i.e. the group with current delusions gave higher ratings for disconfirmed (lure) interpretations and lower ratings for confirmed (true) interpretations. Overall the authors suggest that difficulties in integration of evidence (both confirmatory and disconfirmatory) may be important in understanding the development and maintenance of delusions. The further question of specificity of BADE to delusions has been challenging to answer, with inconsistent findings, likely to reflect at least in part the variability in terms of task and sample selection. The only available meta-analysis (McLean et al., 2016), concluded in favour of a specific association with delusions (with a small effect size). However a number of the included studies (total n = 8) may suffer from methodological weaknesses in not being a priori designed to address this question and the adequacy of the methods used to determine group allocation (particularly in identifying comparison groups of individuals with schizophrenia but without current delusions; see also So et al., 2016).

In addition to the methods and tasks discussed here to assess Belief flexibility and BADE, a number of self-report measures have been designed to tap overlapping constructs. In particular, the Beck Cognitive Insight Scale (Beck et al., 2004), defines cognitive insight as an ability to distance from distorted beliefs and misinterpretations, reappraise them, and to recognize erroneous conclusions. It comprises two domains: ‘Self-Reflectiveness’, captures the willingness to acknowledge fallibility, consider alternate explanations, and recognize dysfunctional reasoning while ‘Self-Certainty’, taps overconfidence in current beliefs and judgments. Similarly the Cognitive Biases Questionnaire (Daalman et al., 2013) and the Davos Assessment of Cognitive Biases Scale (DACOBS; (van der Gaag et al., 2013) include self-report items related to reasoning biases. These self-report questionnaires assess self-awareness of and, in some instances, a preference for certain reasoning processes. Studies employing these measures are generating findings of interest, e.g. in terms of relationships with ‘insight’ in psychosis (O'Connor et al., 2017). However we restrict our focus in the present paper to reasoning assessed ‘in action’ rather than by self-report, given that many of the processes we are considering here operate at least partly outside of conscious awareness (Evans and Stanovich, 2013).

3. Thinking fast and slow: two process models of reasoning
  Within cognitive psychological theory, dual process models of human reasoning posit two parallel systems or processes underpinning decision-making, involving the following key distinction:
  *Type 1: fast, high capacity, independent of working memory and cognitive ability*
  *Type 2: slow, low capacity, heavily dependent on working memory and related to individual differences in cognitive ability.* (see e.g. Epstein, 1994, Evans and Over, 1996, Evans and Stanovich, 2013, Kahneman, 2011, Stanovich and West, 2000)
  Kahneman (2011) popularised this distinction in his book ‘Thinking, fast and slow’. It is apparent that JTC may reflect the operation of Type 1 fast processes while belief flexibility (i.e. an ability to step back, consider the possibility of being mistaken and reflect on alternative explanations) overlaps substantially with the construct of analytic, controlled ‘Type 2’ reasoning. Epstein's Cognitive-Experiential Self-Theory (CEST) and associated dual process nomenclature of ‘experiential/intuitive’ (emotion based) and ‘rational’ systems (Epstein, 1994, Epstein et al., 1996) also adopts this key distinction although important theoretical differences exist within those advocating dual-process theories and Evans (2011) has argued for a shift from a ***systems*** view to a focus on dual ***processes***. In a joint paper, Evans and Stanovich (2013) have articulated a *Default interventionist* position which is consistent with the heuristics and biases research programme of Kahneman (e.g. (Kahneman, 2011, Tversky and Kahneman, 1974). Default Interventionism is the view that reasoning and decision-making sometimes requires both (a) an override of the default (type 1) intuition and (b) its replacement by effective Type 2, reflective reasoning. The issue of ‘override’ has clear relevance to the construct of belief flexibility as applied to delusional thinking, which was originally conceptualised explicitly as an override process, comprising reviews of more rapid and immediate judgments. Evans has further argued for the potential need to distinguish processes that are responsible for resource allocation and conflict resolution between types 1 and 2 thinking (Evans, 2009). These processes serve a similar function to Stanovich's (2009) ‘reflective mind’ which itself follows on Daniel Dennett's (1996) ‘Kinds of minds’ theory (outlining functioning of the autonomous, algorithmic and reflective minds). Default Type 1 processing is viewed as the exclusive domain of the autonomous mind while the reflective and algorithmic minds play separate roles within type 2 processing. The reflective mind has a higher order regulatory function and is related to ‘thinking dispositions’, while the algorithmic mind is associated with individual differences in cognitive ability e.g. fluid intelligence and working memory (Stanovich, 2009). A key putative operation of the reflective mind is to send out a call for the algorithmic mind to engage in hypothetical thinking. To enact hypothetical thinking, the algorithmic mind engages an initial process of cognitive ‘decoupling’ (i.e. generating and sustaining a secondary representation), which is effortful, commanding cognitive resources, and loading heavily on working memory (Evans and Stanovich, 2013).

## 4 Fast and slow thinking: an integration

**Fig. 1:**
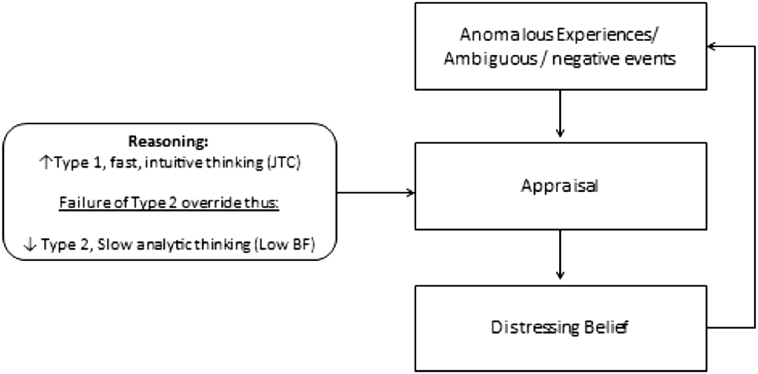
A schematic representation of ‘Thinking, fast and slow’ as it applies to distressing beliefs about others (paranoia).

The evidence summarised above on JTC and data gathering, belief flexibility and evidence integration points convincingly to the presence of characteristic reasoning biases in psychosis and delusions. However, the literature on JTC and the different ways of conceptualising belief flexibility in psychosis are currently disparate, both theoretically and empirically. Dual process models, with the constructs of thinking, fast and slow, popularised by Kahneman (2011), offer scope for integrating the literature on JTC (an aspect of fast thinking) with that on belief flexibility and BADE, which can clearly be construed as the failure of the activation and/or the effective operation of ‘slow’ thinking. This work therefore brings us full-circle given the early influence of the work by Tversky and Kahneman (1974) on the construct of belief flexibility and the development of the MADS, and offers important opportunities both in terms of clinical practice and theory development. We have found that this distinction of thinking fast and thinking slow is a heuristic with clear face validity and readily comprehensible to clinicians and individuals with psychosis alike. In this view, reasoning in the context of distressing delusions might involve an over-reliance on the autonomous mind (i.e. type 1 ‘fast thinking’ including JTC), together with a reduced ‘call to hypothetical thinking’ (type 2) from the reflective mind (involving, for example, a reflective mind preference for intuitive rather than analytic thinking) combined with deficits in the algorithmic mind associated with working memory (i.e. difficulties in sustaining dual representations, generating novel ideas and engaging in mental simulation/thought experiments), ultimately manifesting the reduced belief flexibility noted above. Put more simply, an over-reliance on fast Type 1 reasoning processes together with a reduced likelihood of the activation of override by slow Type 2 processes, provides the context within which the distressing beliefs are maintained and even strengthened over time (see Fig. 1). This figure represents a simple schematic representation to show how dual-process reasoning and reasoning biases may influence appraisals within more comprehensive cognitive models of psychosis (Garety et al., 2001, Garety et al., 2007, Morrison, 2001, Freeman et al., 2002)). Readers are directed to descriptions of these models for fuller accounts of the complex interactions of such cognitive factors with other psychological, social and biological factors in the formation and maintenance of psychosis.

Researchers have started to apply dual process frameworks to examine reasoning across the psychosis continuum. In studies with non-clinical populations, modest positive correlations have been found between experiential reasoning and paranormal and superstitious beliefs and schizotypy, with rational reasoning showing the converse relationship (Aarnio and Lindeman, 2005, Freeman et al., 2012, Wolfradt et al., 1999). In an early study, Freeman et al. (2012) found that a perceived reliance on experiential reasoning is associated with paranoid thinking in the general population, while reliance on deliberation (rational reasoning) is associated with fewer paranoid thoughts. A follow-up to this study (Freeman et al., 2014) replicated the association within a large non-clinical group (n = 1000) between rational reasoning and reduced paranoia but not between experiential reasoning and increased paranoia. It should be noted that most studies in this area have, to date, used self-report measures of dual-process reasoning, most commonly the Rational-Experiential Inventory (Epstein et al., 1996), since there have been no suitable in vivo methods for assessment. Such methods may however be limited by these processes operating outside conscious awareness, as we noted above. Indeed the study by Freeman et al. (2014) found that patients with delusions (n = 30) reported lower levels of *both* experiential and analytic reasoning than the non-clinical individuals (effect sizes small to moderate) and they proposed this might relate to reduced confidence in, or awareness of, reasoning processes within the clinical group. Recently Ward et al. (2017), therefore developed a method of rating dual-process reasoning in vivo and used this to examine explanations of anomalous experiences in people with psychotic experiences with vs. without a ‘need for care’. It was found that marked observed use of experiential reasoning was associated with the presence of psychotic experiences in both groups while higher levels of active rational reasoning processes, together with an absence of JTC, emerged as a potential protective factor against the development of need-for-care in the context of persistent psychotic experiences. Adopting a different approach involving a deductive reasoning task, Speechley et al. (2010) found that individuals with delusions fail to use conflict to modulate towards ‘Stream 2’ (equivalent to Type 2 reasoning) when two streams of reasoning arrive at incompatible judgments, proposed as preliminary evidence of a Dual-Stream Modulation Failure model of delusion formation and maintenance. The findings of these clinical and non-clinical studies, despite being few in number and at an early stage of methodological development, provide some supporting evidence for the relevance of dual-process models in psychosis; in particular suggesting that slower, analytic reasoning may be protective against the development of paranoia while over-reliance on fast, experiential (emotion-based) reasoning may be associated with unusual (delusional) beliefs and paranoia across the psychosis continuum.

## 5 Clinical implications

What are the clinical implications of this strong evidence for a relationship between reasoning biases and distressing delusional beliefs? Relevant to this question are the findings that while JTC predicts change in acute (Menon et al., 2008, So et al., 2014) and first episode (Dudley et al., 2013) psychosis, both JTC and belief flexibility have been shown to remain unchanged by standard CBT or by medication (Brakoulias et al., 2008, Garety et al., 2008, Menon et al., 2008, So et al., 2012, So et al., 2010). Furthermore, an early finding that has since been replicated is that presence of belief flexibility predicts change in response to cognitive behavioural therapy for psychosis (CBTp) i.e. more change occurs if the person has some flexibility; a finding that extends to response to medication (Brett-Jones et al., 1987, Chadwick and Lowe, 1990, Sharp et al., 1996, Garety et al., 1997, So et al., 2012). As well as being a predictor of outcome, belief flexibility has been found to mediate change in paranoia (Garety et al., 2015). Overall these findings suggest that while current psychological therapies are not adequately tackling these reasoning biases, they remain as treatment targets. This is important because it provides the rationale for a ‘causal-interventionist’ approach to improving therapy effectiveness, which involves developing tailored interventions to target the specific mechanisms that research has shown to play a causal role in the problem to be treated (Freeman, 2011, Freeman et al., 2016, Mehl et al., 2015).

In this context, systematic attempts to ameliorate reasoning in people with psychosis have started to emerge internationally, in particular group-based metacognitive training (MCT; Moritz et al., 2013) developed in Germany, with a strong focus initially on JTC as a key reasoning bias. More recently MCT has been expanded to target a broader range of reasoning biases and also to add individual training sessions, MCT +, with some encouraging results for delusion change (Eichner and Berna, 2016, Moritz et al., 2014). However, the two largest RCTs of group MCT have not demonstrated consistent changes in reasoning (Moritz et al., 2013, van Oosterhout et al., 2014). A recent RCT of individualised MCT (MCT +) also found limited evidence of change in thinking processes and some improvements in delusions which were not, however, sustained at follow up (Andreou et al., 2017). Building on Moritz and colleagues' important work, and the literature reviewed in this paper, we have developed a new therapeutic intervention which aims to enhance the impact on thinking processes, by intensively targeting JTC and belief flexibility, in a series of iterations (Garety et al., 2015, Ross et al., 2011, Waller et al., 2015, Waller et al., 2011) finally leading to SlowMo, a protocolised individual therapy for distressing beliefs about harm from others. A pilot feasibility RCT of the version prior to SlowMo found good effect sizes in both improved reasoning processes and reductions in distressing paranoia (Waller et al., 2015), while an associated study demonstrated that changes in paranoia were mediated by belief flexibility (Garety et al., 2015).

**Fig. 2:**
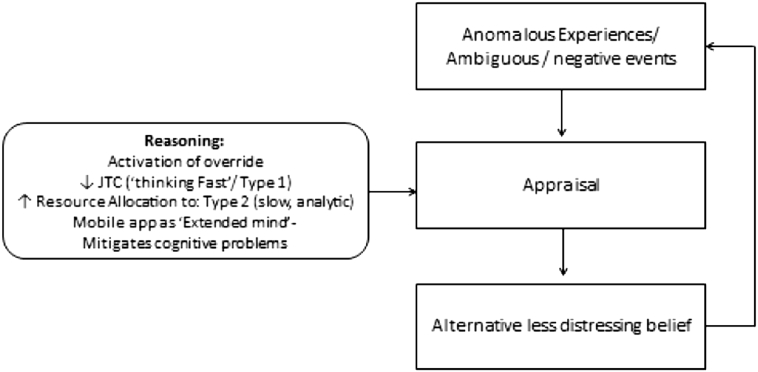
The dual-process treatment rationale of SlowMo therapy-activation of the override of default type 1 (fast) thinking by Type 2 (slow, analytic thinking).

SlowMo is a novel digital therapy, which now explicitly adopts Kahneman's normalising heuristic of ‘thinking, fast and slow’ in order to target the reasoning biases implicated in paranoia (specifically JTC and belief flexibility). The therapy consists of eight individual, face-to-face sessions, delivered by trained therapists, assisted by a website with interactive personal accounts and exercises. Initial sessions involve building the meta-cognitive skill of noticing thoughts (visualised as spinning bubbles) and thinking habits. There is an emphasis on delivering normalising messages regarding the prevalence of fast thinking and worries about others in the general population. People learn that while everyone thinks fast at times and this can be useful, thinking slow can be helpful in dealing with stress and worries about other people. This key principle frames the sessions where people are supported to try out tips to *slow down for a moment*, e.g. by considering the impact of mood and past experiences on worries and looking for safer alternative explanations. Personalised session content (including a visual formulation screen created by the person) is synchronised with a mobile app to assist therapy generalisation into daily life. The mobile app has been designed to optimise type 2 thinking, offering a real-time ‘default-intervention’ on the rapid type 1 reasoning, characteristic of paranoia (see Fig. 2). It is hoped that the app may in some ways act as a kind of ‘extended mind resource’, which encourages allocation of resources to type 2 processing (a key function of the reflective mind) while also reducing the cognitive load on the algorithmic mind by providing ready access to a repository of alternative safety ideas, previously generated in therapy sessions (in the form of easily accessible alternative explanations and safer thoughts). This novel digital therapy has been developed in collaboration between service users, designers, researchers and clinicians. We have recently commenced a large-scale randomised controlled trial, see http://slowmotherapy.co.uk/ which aims to test the efficacy of this therapy for paranoia and also to test the hypothesised mechanism that it works by helping people to slow down their thinking i.e. by making greater use of flexible, type 2 thinking.

## 6 Areas for future research

Reasoning and psychosis is an active and productive research field, generating new theoretical and clinical developments. Much more however remains to be systematically explored. We propose three priorities:

1. Understanding the relationship between reasoning biases and neurocognition
  Elucidating the role of neurocognition in clinical psychosis and specifically its relationship with reasoning is an important but complex issue. We would argue that to date there is a lack of well-powered research using adequate assessment batteries designed to assess the role of cognitive capabilities and deficits in these aspects of reasoning. Little if any work in this area involving belief flexibility (and indeed dual process reasoning) related to psychosis has been conducted, while BADE task findings have been inconsistent. Buonocore et al. (2015) as part of a randomised trial looking at the effect of combining group-based MCT with cognitive remediation therapy (CRT), reported significant correlations between BADE baseline performances and several cognitive domains associated with the frontal executive functions (including cognitive flexibility, working memory and Verbal Fluency). However BADE improvements over time were not associated with improvement in neurocognitive performance (or indeed psychopathology), which was taken as evidence of differential treatment effects for the MCT and CRT. While Riccaboni et al. (2012) also reported correlations between BADE performance and executive functions Moritz et al. (2010) found no correlation between BADE scores and performance on the Trail-making Task (an assessment of executive functioning) with the conclusion that BADE reflects inflexibility towards beliefs rather than problems with set-shifting. With regards to JTC, initial attempts to address this issue have been conducted (Garety et al., 2013a) with the results suggesting that this bias is associated with impairments in working memory (as opposed simply to global impairment in IQ), which can be understood in terms of deficits related to the functioning of the algorithmic mind in the discussion above (Section 4). Other studies where the role of neurocognition (most commonly IQ pro-rated from a short assessment) is analysed commonly adopt ANCOVA or related methods, with some reporting that the contribution of JTC to delusions becomes non-significant after controlling for intelligence (see for example Van Dael et al., 2006). It should be noted that these commonly used statistical methods have been questioned as a valid method for attempting to ‘equalise’ pre-existing groups on real group differences such as those likely to be observed for example in IQ between psychosis groups and healthy controls (for discussion of this issue see Miller and Chapman, 2001). Therefore we would recommend that future research in this important area is carefully designed both in terms of the assessment battery and the statistical approach.
2. The role of social processes and context in reasoning
  The relevance of the literature on reasoning and decision making has been recognised from the early work on the development of the MADS (Wessely et al., 1993). However there has been less consideration of dual-process theory as applied to social processes, despite its importance within the domain of social psychology (see e.g. Bargh and Williams, 2006, Chaiken and Trope, 1999). Potentially important interactions between reasoning and social processes are suggested by the finding of Jolley et al. (2014) that individuals with psychosis with caregivers were nearly three times more likely to show flexibility and five times more likely if the caregiving relationship was characterised by ‘low expressed emotion’. A paradox within psychosis is that although the individual may encounter extreme social isolation and exclusion in daily life, the experiences themselves can be viewed as fundamentally social in nature. While there has been increasing interest in considering the social communication inherent in voice-hearing (see Bell, 2013, Deamer and Wilkinson, 2015, Wilkinson and Bell, 2016, Woods et al., 2014) along with a new wave of explicitly relational therapies (Corstens et al., 2012, Craig et al., 2015, Hayward et al., 2009, Leff et al., 2014), there has been a lesser focus in the context of paranoia despite the typically social themes and apparent involvement of the representation of ‘social agents’. Indeed, recently a so-called Argumentative Theory of human reasoning (Mercier, 2016, Mercier and Sperber, 2011) has proposed that the main function of all reasoning is to exchange arguments with others i.e. reasoning (with all of its inherent heuristics and biases) can be viewed as a fundamentally social exercise. Re-connecting the literature on psychosis and the dual process model of reasoning with general social-cognitive theories may lead to benefits for the understanding of reasoning in psychosis as a social or relational process and reciprocal benefits for general theories of social cognition (Bell et al., 2017) with important implications for research and for psychological and indeed social interventions.
3. Delineating cognitive operations underlying reasoning biases
  Psychosis involves complex multi-modal phenomena and reasoning in the context of paranoia is likely to involve a complex interplay of cognitive, emotional and social and biological processes competing and combining to produce observed behaviour. Attempts to connect different levels of explanation are likely to benefit from a more fine-grained understanding of the cognitive processes underlying task performance of reasoning in action. Lacking from our account at this stage is consideration of the potentially important role of emotion regulation which has also been formulated within a dual-process framework involving a distinction between implicit and explicit emotion processing (see for example Gyurak et al., 2011). Employing a dual process model provides a helpful theoretical framework which integrates the disparate psychosis research findings discussed above. This will also be important for the development of targeted approaches to reasoning biases. Further work in this area would also inform an understanding of who might be more likely to benefit from such approaches (i.e. the personalisation question). Assessment of individual differences in both preferences for the dual process reasoning styles and underlying cognitive operations (e.g. working memory and executive functioning) may aid in tailoring approaches to the needs and abilities of the individual. It may also help in understanding the optimal conditions for activating the reflective mind, which allocates resources to type 2, slower thinking, and whether this can be facilitated in the flow of everyday life for example by the use of digital technology (such as the SlowMo app).

## 7 Conclusion

There is now compelling evidence that reasoning biases are implicated in psychosis and particularly important in the development and maintenance of delusions including paranoia. We have presented evidence on the key biases relating to reasoning ‘in action’ and proposed that these can be understood within Kahneman's distinction between ‘thinking, fast and slow’. This framework has clear clinical utility and affords opportunities for engagement with a broader theoretical terrain in the field of decision-making, reasoning and social psychology. It is also informing a new wave of targeted treatments. We have introduced one such targeted approach, our own SlowMo therapy, which uses digital technology to target JTC and belief flexibility in an attempt to facilitate slower, more reflective thinking when people need it most – in the flow of their daily life.

## Author disclosure

Both authors were fully involved at all stages in the writing of this manuscript.

## Conflicting interests

The authors declare that they have no competing interests.
